# Spontaneous Pneumomediastinum due to Anti-Melanoma Differentiation-Associated Protein 5 Requiring a Bilateral Lung Transplant

**DOI:** 10.1155/2021/6097183

**Published:** 2021-10-29

**Authors:** Amrit Singh Jhajj, James Hok Shun Yeung, Fergus To

**Affiliations:** ^1^Department of Medicine, University of British Columbia, Vancouver, Canada; ^2^Division of Rheumatology, University of British Columbia, Vancouver, Canada

## Abstract

Anti-melanoma differentiation-associated protein 5 (anti-MDA5) is a subset of dermatomyositis associated with respiratory complications, in which rapidly progressive interstitial lung disease (RPILD) is commonly cited, and spontaneous pneumomediastinum (SPM) is a rare complication. In medical literature, aggressive immunosuppressive therapy has been the mainstay of anti-MDA5-associated SPM management. Here, we report the first MDA5 case with SPM which was successfully treated with a double-lung transplant. We present a 48-year-old male who presented with multiple constitutional symptoms such as fevers, weight loss, malaise, and arthralgias, in association with erythroderma over the ears and fingers. Imaging of the chest demonstrated peripheral airspace disease, and myositis-specific serology returned positive for anti-Jo1 (medium-positive), anti-Ro52 (high-positive), and anti-MDA5 (weak-positive) autoantibodies. Therefore, the patient was begun on immunosuppressive therapy as the leading diagnosis included autoimmune myositis, possibly antisynthetase syndrome with interstitial lung disease (ILD). A year later, the patient presented with progressive shortness of breath, widespread macular erythematous facial rash, and new erythematous ulcerations over the fingertips. Imaging demonstrated a new SPM at this juncture. As the patient's respiratory status continued to decline despite the use of immunosuppressive agents, a double-lung transplant was performed. Therefore, we propose that lung transplantation should be considered early in MDA5-SPM.

## 1. Introduction

Autoantibodies (Abs) are increasingly recognized for their pivotal role in the diagnosis and prognosis of inflammatory myopathies. Dermatomyositis can be subclassified according to particular Abs, one of which is anti-melanoma differentiation-associated protein 5 (anti-MDA5), seen in 10–35% of cases [1]. Anti-MDA5 is reported to be an independent risk factor for interstitial lung disease- (ILD-) associated mortality [2]. Although typically associated with clinical amyopathic dermatomyositis (CADM), 42.9–54.5% anti-MDA5 patients can demonstrate overt clinical myopathy [1, 3]. Anti-MDA5 is associated with respiratory complications such as rapidly progressive interstitial lung disease (RPILD) and, as a rare sequalae, spontaneous pneumomediastinum (SPM) [4, 5]. SPM is characterized as free air around the mediastinal structures with an associated prevalence of 2.2% in all myositis cases and a striking mortality rate of 25% within the first month of diagnosis [6]. In this report, we describe the first anti-MDA5 case with SPM which was successfully treated with a double-lung transplant.

## 2. Case Presentation

A 48-year-old diabetic male presented in May 2019 with cough, low-grade fevers, erythroderma over the ears and radial aspect of his fingers (without mechanics hands), 20-pound weight loss, and arthralgias. Blood work was remarkable for a ferritin of 2100 ug/L (normal 15–300 ug/L), ALT of 83 U/L (normal < 55 U/L), LDH of 302 U/L (normal 90–240 U/L), CK of 245 U/L (normal < 165 U/L), and positive anti-scl70 Abs 177 U/mL (normal < 100 U/mL). CT imaging demonstrated patchy multifocal areas of peripheral airspace disease in the lungs with moderate fatty liver infiltration. Prednisone was initiated empirically at 65 mg daily. A week later, a bronchoscopy was performed and was unremarkable. At this time, the differential diagnosis included systemic sclerosis versus relapsing polychondritis, and therefore, prednisone (50 mg daily) and azathioprine (150 mg daily) were begun.

**Figure 1:**
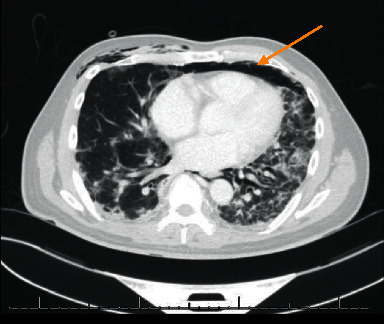
Lung CT scan demonstrating SPM (arrow) prior to lung transplant.

**Figure 2:**
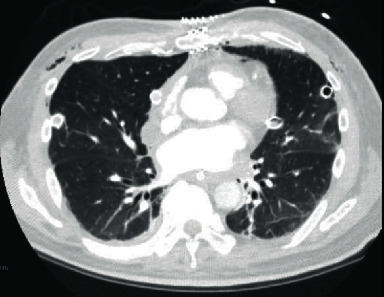
Lung CT scan after bilateral lung transplant.

A month later, as prednisone was tapered down to 10 mg daily, the patient experienced increasing respiratory symptoms with new proximal lower limb muscle weakness. CK was 801 U/L (normal < 165 U/L), and MRI imaging demonstrated edema in the hip and shoulder girdles. Serology for myositis-specific and -associated Abs using line blot immunoassay (EUROLINE Inflammatory Myopathies 16Ag (IgG) (Euroimmun, Lubeck, Germany)) was positive for anti-Jo1 (medium-positive), anti-Ro52 (high-positive), anti-MDA5 (weak-positive), and anti-NT5c1A Abs (no titres provided by assay). Given these findings, autoimmune myositis was considered, possibly antisynthetase syndrome with ILD given the anti-Jo1 positivity. The weakly positive anti-MDA5 was of unclear significance at that juncture. Treatment was modified to prednisone 65 mg daily with taper, mycophenolate 1 g twice daily (uptitrated to 1.5 g twice daily), and hydroxychloroquine 300 mg daily. By August 2019, no myopathy was detected on electromyography and prior skin lesions had improved. In June 2020, he presented with progressive shortness of breath and widespread macular erythematous rashes over his face, with new erythematous ulcerations over the fingertips. MDA5 dermatomyositis was then diagnosed as his clinical phenotype was more in keeping of that than antisynthetase syndrome. A CT scan found extensive subcutaneous emphysema within the upper chest and extensive contiguous pneumomediastinum (Figure 1). IV methylprednisolone (1 gram daily for 3 days) and antibiotics were started, hydroxychloroquine was continued, and mycophenolate was held. His oxygen demands climbed to 55% FiO_2_. His respiratory status continued to decline despite rituximab 1 gram IV, and he went on to require intubation with extracorporeal membrane oxygenation (ECMO). A double-lung transplant was performed 2 days later (Figure 2).

Postoperatively, he was able to taper off oxygen and has remained on room air since. On recovery, investigations have demonstrated the presence of critical illness myopathy without any active myositis. He is currently undergoing intensive inpatient rehabilitation with positive interim results. He remains on tacrolimus 3.5 mg in AM and 3 mg in PM, mycophenolate 1 gram twice daily, and prednisone 15 mg daily for post-lung-transplant therapy.

## 3. Discussion

While there are case reports of lung transplant rescue therapy in MDA5-RPILD (Table 1), to our knowledge, this is the first published case of MDA5-SPM successfully treated with a double-lung transplant. Predictors of poor outcomes in SPM, apart from ILD, include cutaneous vasculopathy, elevated ferritin, anti-Ro-52, and anti-MDA5, factors that were all present in our case [7–9]. The association of clinical variables such as cutaneous vasculopathy and ferritin rise could suggest either an underlying endothelial inflammatory process or coagulation-fibrinolytic system mounting a response in the pathogenesis of SPM-related DM, both of which may guide future therapeutic development [5, 9, 10].

This case also highlights the diagnostic dilemma of managing of concurrent myositis specific autoantibodies (MSAs). Both anti-MDA5 and anti-Jo1 Abs are MSA, which are noted to be exclusive in 99.8% of cases and carry 91.9% false positive rate when only weakly positive [11, 12]. As such, given anti-MDA5 was only weakly positive, the anti-Jo1 was felt to be the true positive initially, leading a working diagnosis of antisynthetase syndrome with ILD. The eventual change in his clinical presentation makes it unlikely that this patient's disease, including ILD, is due to antisynthetase syndrome. His ulcerative cutaneous lesions are much more commonly detected in anti-MDA5 dermatomyositis [13]. Moreover, the speed of this patient's ILD progression is much more characteristic of anti-MDA-5 than antisynthetase syndrome [14]. The anti-MDA5 MSA result represents a true positive, rather than the anti-Jo1, albeit only weakly positive. Therefore, it is imperative that complications of all MSA be monitored early, regardless of strength, as even a concurrent, weakly positive anti-MDA5 Ab ultimately led to bilateral lung transplantation.

Prompt diagnosis and intervention is crucial in MDA5-SPM, as mortality in anti-MDA5-positive cases with and without SPM has been reported to be 60% and 37%, respectively [15, 16]. Early aggressive immunosuppressive therapy and ECMO have been the mainstay treatment of MDA5-SPM. Supplementary ventilation options such as noninvasive positive pressure ventilation (NPPV) are futile given NPPV has been shown to aggravate mediastinal emphysema [15]. In one MDA5-SPM study, mortality was 100% in patients treated with NPPV in comparison to 49% in patients treated without NPPV at 100 days [15]. SPM likely occurs through the Macklin effect of alveolar rupture with retrograde air dissection along the bronchovascular sheath towards the mediastinum. As such, NPPV likely exacerbates the inappropriate outflow of air [9, 15]. Therefore, we propose that, similar to RPILD, lung transplantation should be considered early on in the management of MDA5-SPM.

## Figures and Tables

**Table 1:** Lung transplant rescue therapy in MDA5-RPILD case reports.

Reference	Country	Patient demographics	Clinical features	Positive biomarkers	Outcome
Deitchman et al. [16]	USA	51-year-old male	Small joint arthralgias, cough, dyspnea	Anti-MDA5	Bilateral lung transplant
Huang et al. [1]	Canada	52-year-old male	Heliotrope rash, Gottron's papule, periungal erythema	Anti-RO52, anti-MDA5	Bilateral lung transplant
Huang et al. [1]	Canada	54-year-old female	Heliotrope rash, Gottron's papule, cutaneous ulcers	Anti-RO52, anti-MDA5, OJ	Bilateral lung transplant
Huang et al. [1]	Canada	59-year-old female	Palmar papules, periungal erythema	Anti-RO52, anti-MDA5	Bilateral lung transplant
Leclair et al. [8]	USA	38-year-old male	Heliotrope rash, Gottron's papules, periungal erythema, buccal mucosa erosions, mechanic's hands, arthralgia, exertional dyspnea	Anti-RO52, anti-MDA5	Bilateral lung transplant
Takada et al. [17]	USA	42-year-old female	Rash to the face and hands, leg weakness, dyspnea	Anti-smooth-muscle antibody, serum IgG,	Bilateral lung transplant
Pacot et al. [7]	France	51-year-old male	Acute dyspnea	Anti-MDA5, cytoplasmic islets	Bilateral lung transplant
Shoji et al. [18]	Japan	52-year-old female	Gottron's papules, dyspnea	Anti-MDA5	Bilateral lung transplant

## Abbreviations

Abs: Autoantibodies
Anti-MDA5: Anti-melanoma differentiation-associated protein 5
ILD: Interstitial lung disease
CADM: Clinical amyopathic dermatomyositis
RPILD: Rapidly progressive interstitial lung disease
SPM: Spontaneous pneumomediastinum
CT: Computerized tomography
CK: Creatinine kinase
LDH: Lactate dehydrogenase
ALT: Alanine aminotransferase
ECHMO: Extracorporeal membrane oxygenation
NPPV: Noninvasive positive pressure ventilation
MSA: Myositis specific autoantibodies.
